# Preclinical HER-2 Vaccines: From Rodent to Human HER-2

**DOI:** 10.3389/fonc.2013.00151

**Published:** 2013-06-10

**Authors:** Pier-Luigi Lollini, Carla De Giovanni, Patrizia Nanni

**Affiliations:** Laboratory of Immunology and Biology of Metastases, Department of Experimental, Diagnostic and Specialty Medicine, Alma Mater Studiorum University of Bologna, Bologna, Italy

**Keywords:** HER-2, tumor immunology, oncoantigens, mouse models, metastasis, immunodeficient mice

## Abstract

Effective prevention of human cancer with vaccines against viruses, such as HBV and HPV, raises the question whether also non-virus related tumors could be prevented with immunological means. Studies in HER-2-transgenic mice showed that powerful anti-HER-2 vaccines, could almost completely prevent the onset of mammary carcinoma. Protective immune responses were orchestrated by T cells and their cytokines, and effected by antibodies against HER-2 gene product p185. Analogous findings were reported in a variety of other cancer immunoprevention systems, thus leading to the definition of oncoantigens, optimal target antigens that are causally involved in carcinogenesis and cancer progression. Prophylactic HER-2 vaccines were also effective in preventing metastasis outgrowth, indicating that concepts and approaches developed for cancer immunoprevention could prove fruitful in cancer immunotherapy as well. The availability of cancer-prone mice carrying a human HER-2 transgene is now fostering the design of novel vaccines against human p185. A further bridge toward human cancer was recently provided by novel immunodeficient models, like Rag2^−/−^;Il2rg^−/−^ mice, which are permissive for metastatic spread of human HER-2+ cancer cells and can be engrafted with a functional human immune system, allowing for the first time the study of vaccines against oncoantigens to elicit human immune responses against human cancer cells *in vivo*.

## HER-2 is a Class I Oncoantigen

HER-2 is at the same time an oncogene and an antigen. HER-2-addicted cancer cells cannot easily give rise to antigen-loss variants to escape immune attacks (Nanni et al., 2000).

Furthermore, the HER-2 gene product, p185, is a surface protein, hence it is recognized and bound by antibodies. It is also recognized by T cells, as a result of antigen processing and major histocompatibility complex (MHC) presentation. The persistence of recognition by antibodies in the presence of MHC loss makes p185 impervious to the total loss of immune recognition that affects intracellular tumor antigens.

These properties make HER-2 a class I oncoantigen. Oncoantigens are defined as those tumor antigens that have a causal role in tumor growth and progression (Lollini et al., 2006). Recently, the oncoantigens were divided in three classes, according to surface (class I), extracellular (class II), or intracellular (class III) protein localization (Lollini et al., 2011).

More than half a century of intensive immunological research has led to the accumulation of a database of hundreds of molecules defined as tumor antigens. However, their suitability as targets of cancer preventive or therapeutic approaches is uneven. The definition of oncoantigens was a first step to define priorities in the galaxy of tumor antigens, first between oncoantigens and non-oncoantigens, then among oncoantigen classes. The readers interested in a more clinically oriented attempt at tumor antigen prioritization are referred to a recent collective work of tumor immunologists (Cheever et al., 2009). It is interesting to note that two properties taken into account were “oncogenicity” and “cellular location of antigen,” that is the defining properties of class I oncoantigens.

In summary, HER-2 is endowed with relevant immunological properties that many other tumor antigens lack, making it an ideal target antigen for long-term cancer immunoprevention.

## Cancer Immunoprevention

The immune system actively prevents tumor onset, as is clearly demonstrated by the huge increase in cancer development in genetically modified immunodeficient mice. The same happens in immunodeficient humans, who are mostly prone to viral tumors. Altogether, it can be said that the immune system defends the host both from pathogens and from tumors, and that any decrease in the immune defenses exposes the host to the risk of infections and neoplasms (Dunn et al., 2004; Finn, 2008; Cavallo et al., 2011; Hanahan and Weinberg, 2011).

Just as immunity from microbes is not 100% efficient, thus leaving the host exposed to the risk of a variety of infections, immunity from tumors is not perfect, and the risk of carcinogenesis is always present. This is especially true in the aging hosts, owing to a long history of potentially tumorigenic DNA mutations and to the age-related impairment of immune defenses.

A further parallel between infectious and tumor immunity is the possibility to enhance the immunity of healthy individuals with vaccines. In the field of cancer preventive vaccines, we have now two distinct stages of development. The vaccines against oncogenic viruses are already implemented at the population level, to immunize healthy individuals against hepatitis B virus or papillomaviruses (Schiller et al., 2008; Chang et al., 2009). However, most tumors (>80%) are not caused by infectious agents, and no prophylactic vaccine exists to immunize humans against such tumors. We will discuss here the preclinical development of vaccines to prevent carcinogenesis in HER-2-transgenic mice, showing that non-infectious tumors could be prevented with immunological means.

## Mouse Models for Anti-HER-2 Vaccines in Cancer Immunoprevention and Immunotherapy

The earliest studies of cancer immunoprevention were done in HER-2-transgenic mice harboring an activated (i.e., mutant) version of the rat oncogene, which causes a very aggressive mammary carcinogenesis. In these mice, the first carcinoma appears at 4–5 months of age, and is rapidly followed by other primary tumors in all ten mammary glands; most animals develop also distant metastases (Boggio et al., 1998).

Human cancer is often caused by a combination of mutations in oncogenes and tumor suppressor genes. To model this situation, we then applied cancer immunoprevention to a bigenic mouse model that combines HER-2 activation and heterozygous p53 knockout. Female mice develop mammary and salivary carcinomas (Croci et al., 2004). It should be noted that the null p53 allele in itself does not provide immunological targets, therefore, in these bigenic mice, as in HER-2 transgenic mice, p185 is the target antigen.

More recently, cancer-prone transgenic mice lines carrying a human HER-2 transgene were also established (Finkle et al., 2004). In a sense, these mice validate previous models, because mammary carcinogenesis resembles that of mice carrying the rat HER-2 transgene (Guy et al., 1992). The added value of human HER-2 transgenic mice stems from two types of specific application. They can be used for preclinical testing of therapeutic agents against human p185, for example monoclonal antibodies (Finkle et al., 2004) that are not cross-reactive with rat HER-2. A more recent application of human HER-2 expression in transgenic mice was the establishment of mice carrying HER-2 variants found in patients, such as the Delta16 isoform (Castiglioni et al., 2006; Marchini et al., 2011), thus allowing the study of their carcinogenicity and the direct comparison between wild-type and variant or mutant forms of the HER-2 oncogene.

The major clinical issue of HER-2+ human breast cancer is the metastatic spread to distant organs, including the lungs, the bones, the brain, and various abdominal viscera. The study of HER-2+ cancer dissemination has been hindered by the notorious metastatic inefficiency of human HER-2+ breast cancer cell lines in immunodeficient mouse models, such as athymic nude mice. Most studies used HER-2-negative cell lines transfected with the HER-2 gene under the control of a constitutive promoter, however it is questionable whether forced expression of HER-2 in a HER-2-negative cell provides a realistic target for preclinical cancer therapy.

We have recently found a solution to this long-standing problem, thanks to the use of newer immunodeficient mouse hosts. Double knockout BALB/c Rag2^−/−^;Il2rg^−/−^ (BRG) mice lack T, B, and natural killer (NK) cells, and are popular hosts for reconstitution with normal human immune cells. Some years ago we found that BRG mice are superior to nude mice for studies of metastatic dissemination of human sarcomas (Nanni et al., 2010), therefore we compared the metastatic capacity of human HER-2+ breast cancer cell lines MDA-MB-453 and BT-474 in nude and in BRG mice (Nanni et al., 2012).

**Figure 1:**
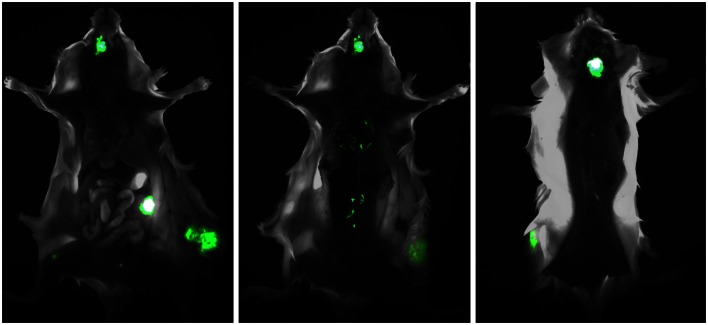
**Multiorgan metastasis of HER-2+ human breast cancer in a BRG mouse**. The intramammary implant of EGFP-transduced MDA-MB-453 cells produced a local tumor which in turn gave rise to multiple metastases in the bones (mandible and femur, *left panel*), the ovary (*left panel*), the lungs (*center panel*), the paravertebral lymph nodes (*center panel*), and the brain (*right panel*). *Left panel*, ventral view; *center panel*, ventral view after removal of the rib cage and abdominal viscera; left panel, dorsal view after partial removal of the brain, to reveal a large underlying metastasis.

We found that, unlike nude mice, BRG mice allow a multiorgan metastatic spread that closely resembles the dissemination patterns of breast cancer in patients. Metastases were found in the brain, the lungs, the liver, the kidneys and adrenals, the ovaries, the bones, and the bone marrow, and in various other anatomical locations (Figure 1). Interestingly, overlapping patterns of metastatic dissemination were observed both after intravenous administration of cancer cells and from local tumors growing in the mammary fat pad or subcutaneously (Nanni et al., 2012).

In the past, the low metastatic potential of malignant human cancers in immunodeficient mice was attributed to xenogeneic incompatibilities between human and murine cognate molecules controlling the metastatic process. Our results in BRG mice clearly demonstrate that, in the case of HER-2+ carcinomas, the major obstacle to metastatic spread was the residual immune response of nude mice, in particular NK activity, and that the full metastatic potential of human cancer can be expressed in BRG mice. This new model offers excellent opportunities for the preclinical testing of antimetastatic drugs. As a first example, we found that BKM120, a PI3K inhibitor that penetrates the blood-brain barrier, effectively inhibited brain metastases of HER-2+ breast cancer (Nanni et al., 2012).

Human hematopoietic stem cells implanted in BRG mice give rise to a functional human immune system, and reconstituted mice can be used for studies of lymphopoiesis and to analyze specific human immune responses (Brehm et al., 2010; Rongvaux et al., 2013). In general, such studies reveal suboptimal human responses, nonetheless this is the first *in vivo* model system allowing the investigation of human immune responses without the human body.

## The Triplex Vaccine, or How to Increase the Immunogenicity of Cancer Cells for Cancer Immunoprevention

Our first attempt at cancer immunoprevention in HER-2 transgenic mice was based on the systemic administration of recombinant interleukin 12 (IL-12), a key cytokine of antigen presentation, that also stimulates NK activity (Boggio et al., 1998). The IL-12 treatment significantly delayed tumor onset, however all mice eventually succumbed to progressive mammary carcinomas. To improve the efficacy of cancer immunoprevention, we decided to switch from a non-antigen specific cytokine to antigen-specific cell-based vaccines.

The major problem of vaccines made of tumor cells is that, in most instances, the immunogenicity of unmodified tumor cells is insufficient to elicit protective immune responses. The history of cell-based cancer vaccines is mostly the history of the approaches and technologies used to increase immunogenicity. Early attempts, based on physical treatments (e.g., heat or UV radiation), chemical modifications, or infections with viruses had limited success (Kobayashi, 1986). A major impulse to this field came from the adoption of gene transduction, to obtain genetically modified tumor cells expressing defined immunogenic stimuli. The use of standardized recipient cell lines, such as the TS/A mammary adenocarcinoma, allowed meaningful comparisons between promising candidate genes, in particular those encoding cytokines (Allione et al., 1994; Musiani et al., 1997). We showed that allogeneic class I MHC genes elicited strong polyclonal T cell activation that could further increase the immunogenicity of cytokine gene-transduced tumor cells (Nanni et al., 1996).

Our previous experience with recombinant cell vaccines for cancer therapy led to the design of the Triplex vaccine for the immunoprevention of mammary carcinoma in HER-2 transgenic mice. Triplex vaccines combine the p185 target antigen with two powerful non-antigen specific stimuli, IL-12 and allogeneic class I MHC molecules. The first implementation of the Triplex concept combined vaccinations with HER-2-transgenic mammary carcinoma cells expressing allogeneic class I MHC molecules and systemic administrations of recombinant mouse IL-12 (Nanni et al., 2001). The same cells were subsequently transduced with IL-12 genes, to obtain local IL-12 release, thus avoiding systemic cytokine administrations (De Giovanni et al., 2004). Both formulations of the Triplex vaccine yielded positive results, that are discussed together in the following sections.

## Immunoprevention of Mammary Carcinoma in HER-2 Transgenic Mice

Vaccination of HER-2 transgenic BALB-NeuT mice with the Triplex vaccines yielded a strong protection from mammary carcinoma onset. All vaccinated mice were still tumor-free at 1 year of age, more than 6 months after all non-vaccinated mice developed progressive carcinomas (Nanni et al., 2001; De Giovanni et al., 2004). The histological and molecular study of mammary glands revealed that, at 1 year of age, vaccinated mice only had hyperplastic lesions similar to those of young mice. In practice, vaccinations had “frozen” progression at a very early stage, as vaccinated mice were also devoid of *in situ* carcinomas (Nanni et al., 2001; Astolfi et al., 2005).

Tumor progression in HER-2-transgenic mice is obviously accompanied by a very high expression of p185 in the mammary epithelium. To our surprise, mammary glands of vaccinated mice expressed very little p185. In practice, the vaccination inhibited the constitutive expression of the activated oncogene in the target tissue (Nanni et al., 2001; De Giovanni et al., 2004).

A complex and powerful vaccine, like the Triplex, entails a large number of experimental variables, in terms of vaccine components and vaccination schedules, therefore we undertook a series of studies to define the relevant components. The vaccination of mice with single or paired components of the vaccine prolonged the latency of mammary carcinomas, but in all cases it failed to keep mice completely free from tumors for 6 months or more, thus demonstrating that the Triplex was the minimal combination affording full cancer prevention for at least a year (Palladini et al., 2010). The use of short, or less intense vaccination schedules led to similar conclusions. In all cases tumor-free survival was reduced with respect to the chronic vaccination schedule (Palladini et al., 2010).

The prophylactic HER-2 vaccines were also effective in preventing metastasis outgrowth, thus indicating that the concepts and the approaches developed for cancer immunoprevention could be effective also in cancer immunotherapy (Nanni et al., 2007).

## Protective Immune Mechanisms Elicited by the Triplex Vaccine

We did not find evidence of strong cytotoxic T cell responses in vaccinated mice. Major long-term effects of vaccination were the induction of T cell cytokines, in particular IFN-gamma, and of antibodies against p185 (Nanni et al., 2004).

To dissect more precisely the protective mechanisms, we crossed BALB-NeuT transgenic mice with knockout mice deficient in IFN-gamma or antibody production. In both cases the Triplex vaccine became ineffective, thus indicating that both IFN-gamma and antibodies were essential components of the protection from mammary carcinogenesis. Interestingly, IFN-gamma deficiency did not impair IgG1 responses to the vaccine, but abolished the production of IG2a, IgG2b, and IgG3, thus indicating that cytotoxic “Th1” immunoglobulin isotypes were the mediators of tumor prevention. In antibody-deficient mice, it was found that IFN-gamma was released in response to the vaccine. This shows that, without antibodies, IFN-gamma does not prevent tumor growth, therefore the key role of Triplex-induced IFN-gamma in this system was to induce the isotypic switch to Th1-type immunoglobulins (Nanni et al., 2004).

The fundamental role of antibodies in tumor prevention allowed us to monitor the protective immune responses during long-term vaccination studies. We found that the kinetics of antibodies elicited by Triplex vaccines predicted, several months in advance, the long-term tumor-free survival of mice. The two major variables related to tumor prevention were a rapid induction of high-titer specific antibodies and a long-term maintenance of the antibody plateau. Any vaccination schedule failing to attain either target inevitably resulted in tumor onset (Palladini et al., 2010).

## Different Vaccines for the Immunoprevention of Mammary Carcinoma in HER-2 Transgenic Mice

The prevention of mammary carcinoma in HER-2 transgenic mice was obtained by many Laboratories, using a variety of immunological approaches, such as cell vaccines (Reilly et al., 2000; Nanni et al., 2001; Murata et al., 2006; Masuelli et al., 2007), exosomes (Hartman et al., 2011), protein or peptide vaccines (Esserman et al., 1999; Dakappagari et al., 2000; Vertuani et al., 2004; Gritzapis et al., 2010; Marzocchella et al., 2011; Uram et al., 2011), DNA and viral vaccines (Amici et al., 1998; Pupa et al., 2005; Tegerstedt et al., 2005; Jacob et al., 2006, 2009, 2010; Whittington et al., 2009; Quaglino et al., 2010), and dendritic cell vaccines (Sakai et al., 2004; Nabekura et al., 2008; Wei et al., 2009; Steel et al., 2010; Landuzzi et al., 2011; Wang et al., 2012). These results, which we recently reviewed in detail (Lollini et al., 2011), indicate that effective immunoprevention of HER-2-driven mammary carcinogenesis can be obtained with different vaccine technologies.

## Immunoprevention of a Polygenic Cancer Syndrome

The Triplex vaccine was highly effective in preventing carcinogenesis also in the HER-2 transgenic, p53 hemizygous mice. Interestingly, the tumors of non-vaccinated mice invariably lacked the wild-type p53 allele and expressed HER-2, whereas the corresponding tissues of vaccinated mice were heterozygous, and showed a low expression of HER-2. The results suggest that, in this system, HER-2 hyperactivity enhanced the risk of p53 loss, and that the vaccine-induced HER-2 suppression prevented also the occurrence of further oncogenic events affecting p53 (Croci et al., 2004).

In HER-2 transgenic BALB-NeuT mice, different Laboratories have produced impressive immunopreventive results using DNA vaccines against HER-2 (Rovero et al., 2000; Pupa et al., 2001; Quaglino et al., 2004; Jacob et al., 2006; Gallo et al., 2007; Cho et al., 2008). We exploited their experience to design a DNA vaccine based on the Triplex concept, that combined plasmids encoding HER-2 extracellular domain, IL-12, and a MHC gene allogeneic with respect to the host (De Giovanni et al., 2009). A direct comparison showed that both vaccine designs were equally effective in preventing tumor onset, with some differences in the elicited immune mechanisms. From a vaccinological perspective, the overall conclusion was that the DNA vaccine, being molecularly defined and requiring a lower number of administrations, had a higher translational potential than the cell vaccine (De Giovanni et al., 2009).

In addition to the bigenic mice discussed here, the concept of cancer immunoprevention was applied in recent years to many different mouse models of cancer risk. We have recently reviewed the subject, which goes beyond the present focus on HER-2, therefore we will not duplicate here the extensive list of references (Lollini et al., 2011). Cancer immunoprevention appears to be generally applicable to the prevention of diverse tumor types, caused by a wide range of cancer genes, using a great variety of effective immunological approaches.

## Preclinical Vaccines Against Human HER-2

The results discussed above were obtained in mice carrying rat HER-2 transgenes. More recently, we found that cell and DNA vaccines against human HER-2, similar in design to those described above, were effective in preventing mammary carcinoma onset in human HER-2 transgenic mice (De Giovanni, manuscript in preparation). The efficacy of anti-human HER-2 vaccines in immunocompetent, human HER-2 transgenic mice shows that these models can be useful in the translation of vaccines from preclinical to clinical situations.

A further step in translational studies is the use of “humanized” mice, i.e., immunodeficient mice reconstituted with a human immune system, to investigate human, instead of mouse, immune responses against HER-2.

We studied the induction of human anti-human HER-2 immune responses in BRG mice reconstituted at birth with human cord blood CD34+ cells (De Giovanni et al., 2012). Young reconstituted mice received a vaccine designed according to the Triplex concept, consisting of HER-2+ SK-OV-3 cells and human IL-12. No attempt was made to match the MHC of donor CD34+ cells with that of vaccine/tumor cells, therefore the cellular component of the vaccine was xenogeneic in relation to the mouse and allogeneic relative to the reconstituted immune system. Immunized mice were then repeatedly bled to study serological responses, and finally challenged with live SK-OV-3 cells to verify protection.

We found that indeed vaccinated mice were partially protected, in particular from metastases. Obviously, the protection could be attributed, in part, to the allogeneic components of the human immune response. However, serological studies revealed the induction of human antibodies against human p185, thus demonstrating that the vaccine was effective in eliciting a specific human immune reaction resembling those previously obtained in rodent systems (De Giovanni et al., 2012).

## Translational Developments

The translation of immunoprevention to HER-2-positive human breast tumors would be a stimulating perspective. Novel strategies to prevent the development of invasive breast cancer are highly desirable for women at risk. For a review of preventive approaches based on HER-targeted drugs or passive immunoprevention, i.e., monoclonal antibodies, see (Howe and Brown, 2011). Immunoprevention of breast cancer could be applied to women at high risk of invasive carcinoma, in particular to those with preneoplastic or early neoplastic conditions, such as atypical hyperplasia or carcinoma *in situ*. It should be noted that the proportion of HER-2-positive ductal carcinomas *in situ* (DCIS) is significantly higher than that of more advanced cases (Muggerud et al., 2010; Zhang et al., 2012). Neo-adjuvant clinical trials of vaccination with HER-2-pulsed dendritic cells have been recently reported (Sharma et al., 2012), thus suggesting that HER-2 vaccines could be applied to HER-2-positive DCIS.

The prevention of HER-2+ metastasis development using the Triplex vaccine (Nanni et al., 2007) indicated that effective anti-HER-2 vaccines could be included in adjuvant therapy of HER-2+ breast cancer, possibly as a strategy to maintain a long-term antibody response to p185 after cessation of the monoclonal antibody regimens.

## Conflict of Interest Statement

The authors declare that the research was conducted in the absence of any commercial or financial relationships that could be construed as a potential conflict of interest.
